# Supplementary material for: Conditional knockout of Shank3 in the ventral CA1 by quantitative in vivo genome-editing impairs social memory in mice
Source: Nat Commun. 2024 Jun 12;15:4531. doi: 10.1038/s41467-024-48430-x (PMC11169449; doi:10.1038/s41467-024-48430-x)
Supplement: Supplementary file 1 — Supplementary Information for Conditional knockout of Shank3 in the ventral CA1 by quantitative in vivo genome-editing impairs social memory in mice [file 41467_2024_48430_MOESM1_ESM.pdf]

Supplementary Information for

Conditional knockout of *Shank3* in the ventral CA1 by quantitative *in vivo* genome-editing impairs social memory in mice

Myung Chung, Katsutoshi Imanaka, Ziyan Huang, Akiyuki Watarai, Mu-Yun Wang, Kentaro Tao, Hirotaka Ejima, Tomomi Aida, Guoping Feng, Teruhiro Okuyama

*Correspondence: Teruhiro Okuyama
Email: okuyama@iqb.u-tokyo.ac.jp

This PDF file includes:

Supplementary Figure 1 to 4
Supplementary Table 1 to 2

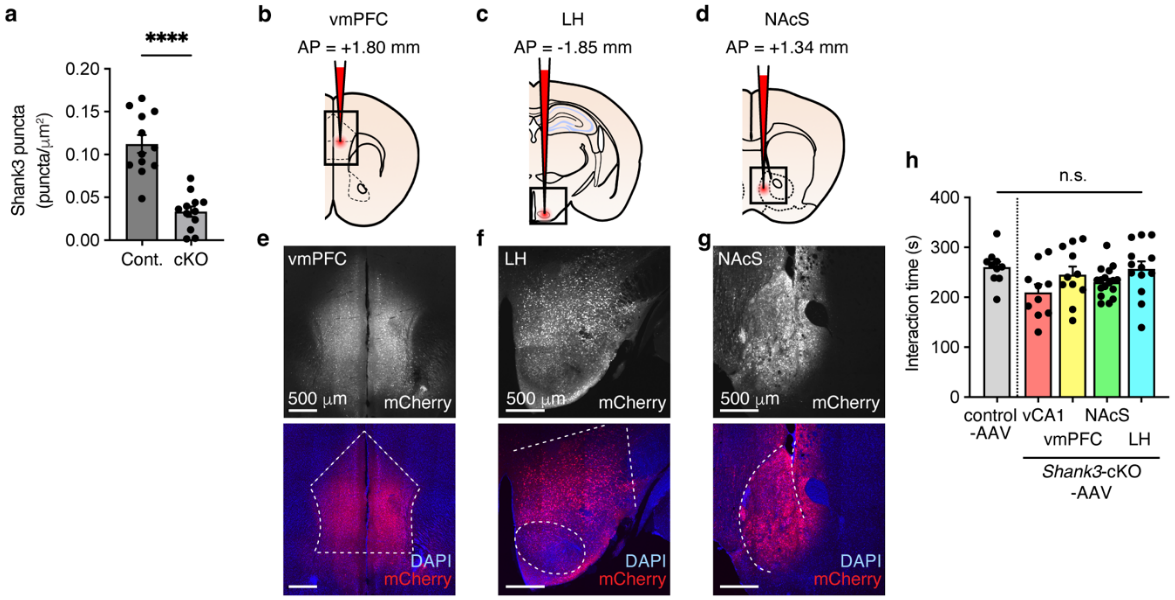

Supplementary Fig. 1. Validation of the AAV-mediated *in vivo* genome editing.

a. The number of Shank3 puncta in vCA1 of control-AAV injected and Shank3-cKO-AAV injected mice (Control AAV: n = 12 sections/4 mice; vCA1-*Shank3*-cKO: n = 12 sections/4 mice, unpaired t-test, two-tailed, t(22) = 6.505, ****p < 0.0001). **b-d.** Schematic illustration of AAV microinjection into vmPFC **(b)**, LH **(c)**, and NAcS **(d)**. **e-g** Representative confocal microscopy images of AAV-injected vmPFC **(e)**, LH **(f)**, and NAcS **(g)** stained with anti-RFP (red, for mCherry) and DAPI (blue). Scale bar = 500 µm. **h** Comparison of the total social interaction time (Novel + Familiar) between the AAV-injected groups (Control AAV, n = 10; vCA1- Shank3-cKO, n = 10; vmPFC-*Shank3*-cKO, n = 11; NAcS-*Shank3*-cKO, n = 17; LH-*Shank3*-cKO, n = 13. One-way ANOVA, F[4,56] = 1.862, p = 0.0670). Data presented as the mean ± SEM. Source data are provided as a Source Data file.

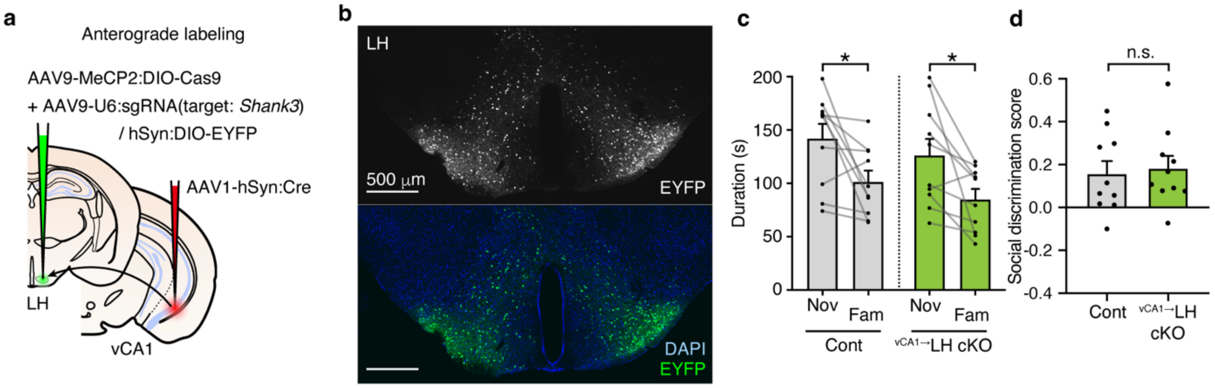

Supplementary Fig. 2. *Shank3*-cKO in the vCA1→LH neurons does not alter social memory.

a. Schematic illustration of viral injection strategy of *Shank3*-cKO in the vCA1→LH neurons. Cre is derived anterogradely from vCA1 to LH, and Cas9 is expressed in a Cre-dependent manner in LH. **b.** Representative microscopic image of LH stained with anti-GFP (green, EYFP) and DAPI (blue). **c**. Social discrimination test of the *Shank3* vCA1→LH cKO mice (Control: n = 10 mice, vCA1→LH cKO: n = 10 mice. Two-way mixed-model ANOVA, Familiarity × Group, $F[1,18] < 0.0001$, $p = 0.9784$; Familiarity, $F[1,18] = 15.99$; ***$p = 0.0008$; Group, $F[1,18] = 1.289$, $p = 0.2712$; Control, $p = 0.0237$; cKO, $p = 0.0162$). **d**. Social discrimination score (Control: n = 10 mice, vCA1→LH cKO: n = 10 mice, unpaired t-test, two-tailed, $t(18) = 0.3006$, $p = 0.7672$). Data presented as the mean ± SEM. Source data are provided as a Source Data file.

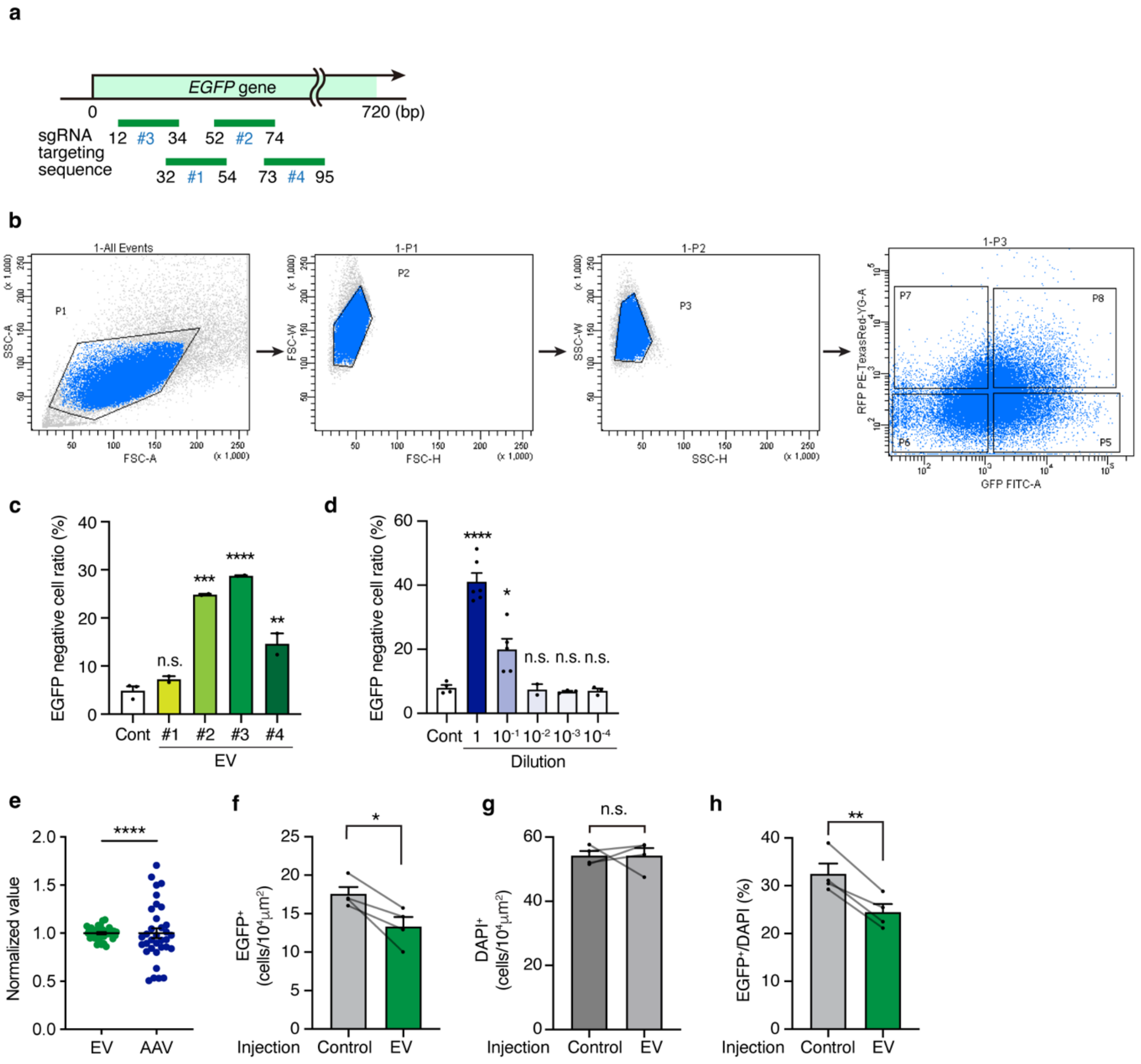

Supplementary Fig. 3. ***In vitro*** **and** ***in vivo*** **application of EGFP gene targeting EV and AAV.** **a**. Schematic illustration of the positions of the EGFP gene-targeting sgRNA on the EGFP gene. sgRNAs #1–#4 were used to produce EV-#1–#4. **b**. Gating strategy for flow cytometry. FSC/SSC gates were used to exclude potential doublet cells. **c**. Quantification of the EGFP-negative cell ratio in the negative control (control-EV) and EGFP-targeting EV (EV-#1–#4)-transduced HEK293-EGFP cells. (Control: n = 3, EV-#1–#4: n = 2. One-way ANOVA followed by Dunnett's multiple comparison test, two-sided. F(4, 6) = 66.82, ****p < 0.0001; Control vs. EV-#1, p = 0.8145; Control vs. EV-#2, ***p = 0.0001; Control vs. EV-#3, ****p < 0.0001; Control vs. EV-#4, **p = 0.0076). **d**. Quantification of the EGFP negative cell ratio of the negative control (control-AAV), undiluted (1), and serially diluted (10^{-1}, 10^{-2}, 10^{-3}, 10^{-4}) *EGFP*-targeting AAV transduced HEK293-EGFP cells (Control: n =4, 1: dilution 1: n = 6, Dilution 10^{-1}: n = 5, Dilution 10^{-2}: n = 2, Dilution 10^{-3}: n = 3, Dilution 10^{-4}: n = 3, One-way ANOVA followed by Dunnett's multiple comparison test, two-sided. F(5, 17) = 34.02, ****p < 0.0001; Control vs. 1, ****p < 0.0001; Control vs. 10^{-1}, *p = 0.0130; Control vs. 10^{-2}, p > 0.9999; Control vs. 10^{-3}, p = 0.9982; Control vs. 10^{-4}, p = 0.9994). **e**. Mean normalized values of the EGFP-negative cell ratio in *EGFP*-targeting EV- and AAV-treated

HEK293-EGFP cells (Fig. 2e, g). EV: n = 36 samples; AAV: n = 34 samples. F-test. $p < 0.0001$. **f-h**. Comparison of the number of EGFP-expressing cells (**f**, n = 4, paired t-test, two-sided, *p = 0.023), DAPI-positive cells (**g**, n = 4, paired t-test, two-sided, $p > 0.99$), and the EGFP+/DAPI+ cell ratio (**h**. n = 4, paired t-test, two-sided, **p = 0.0050) in the control and *EGFP*-targeting EV-injected NAc of Drd1-EGFP mice. *p < 0.05, **p < 0.01, **p < 0.001, ***p < 0.0001. Data presented as the mean ± SEM. Source data are provided as a Source Data file.

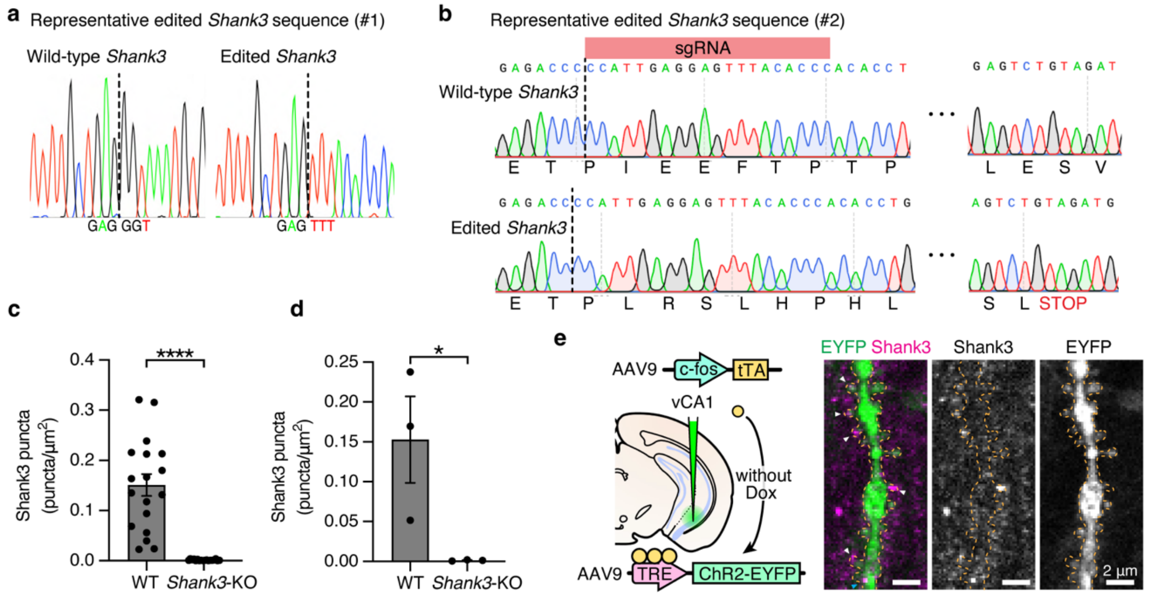

Supplementary Fig. 4. Confirmation of *Shank3*-cKO by EV-mediated methods.
a. Representative wild-type (unedited) and edited *Shank3* sequences (#1). **b.** Representative wild-type (unedited) and edited *Shank3* sequences near the sgRNA sequence (#2). Edited *Shank3* contains a premature termination codon. **c-d.** Shank3 puncta analysis of WT and *Shank3*-KO mice by section (**c**, n = 18 sections each, unpaired t-test, two-sided, ****p < 0.0001) and individual (**d**, n = 3 mice each, unpaired t-test, two-sided, *p = 0.049). **e**. Shank3 puncta in the spine-like structure of the social memory neuron in vCA1. Left, Schematic illustration of the strategy of social memory neuron labeling; Right, Representative microscopic image of a social memory engram neuron stained with anti-GFP (green, EYFP) and anti-Shank3 (magenta, Shank3). White arrowhead, Shank3 puncta at the spine-like structure; Blue arrowhead, puncta in the dendrite. Bar = 2 μm. *p < 0.05, ****p < 0.0001. Data presented as the mean ± SEM. Source data are provided as a Source Data file.

Supplementary Table 1. Statistical information for Figures			
**Figure**	**Sample Size**	**Analysis**	**Statistical Test Values (*p<0.05, **p<0.01, ***p<0.001, ****p<0.0001**
Figure 1g	n = 61 mice<br><br>Control: 10 mice<br>vCA1-cKO: 10 mice<br>vmPFC-cKO: 11 mice<br>NAcS-cKO: 17 mice<br>LH-cKO: 13 mice	Two-way mixed model ANOVA	(Familiarity×Group)<br>F(4,56) = 6.270; ***p = 0.0003<br>(Familiarity)<br>F(1,56) = 21.65; ****p < 0.0001<br>(Group)<br>F(4,56) = 2.331; p = 0.0670<br>(Control)<br>F(1,9) = 23.63; ***p = 0.0009<br>(vCA1-cKO)<br>F(1,9) = 0.103; p = 0.756<br>(vmPFC)<br>F(1,10) = 9.158; *p = 0.013<br>(NAcS-cKO)<br>F(1,16) = 4.649; *p = 0.047<br>(LH)<br>F(1,12) = 0.012; p = 0.913
Figure 1h	n = 61 mice<br><br>Control: 10 mice<br>vCA1-cKO: 10 mice<br>vmPFC-cKO: 11 mice<br>NAcS-cKO: 17 mice<br>LH-cKO: 13 mice	One-way ANOVA<br><br>Scheffe's multiple comparison	F(4,54) = 5.377; **p = 0.0010<br><br>Control vs. vCA1-cKO: **p = 0.0032<br>Control vs. vmPFC-cKO: p = 0.2968<br>Control vs. NAcS-cKO: p = 0.0817<br>Control vs. LH-cKO: **p = 0.0037<br>vCA1-cKO vs. vmPFC-cKO: p = 0.3709<br>vCA1-cKO vs. NAcS-cKO: p = 0.5304<br>vCA1-cKO vs. LH-cKO: p = 0.9989<br>vmPFC-cKO vs. NAcS-cKO: p = 0.9917<br>vmPFC-cKO vs. LH-cKO: p = 0.4637<br>NAcS-cKO vs. LH-cKO: p = 0.6457
Figure 2e	n = 36 samples<br><br>Control: 6 samples<br>1/1: 6 samples<br>1/2: 6 samples<br>1/4: 6 samples<br>1/8: 6 samples<br>1/16: 6 samples	One-way ANOVA<br><br>Dunnett's multiple comparisons test	F(5,30) = 343.4, ****p < 0.0001<br><br>Control vs. 1/1: ****p < 0.0001<br>Control vs. 1/2: ****p < 0.0001<br>Control vs. 1/4: ****p < 0.0001<br>Control vs. 1/8: **p = 0.0028<br>Control vs. 1/16: p = 0.8521
Figure 2g	n = 34 samples<br><br>Control: 4 samples<br>1/1: 6 samples<br>1/2: 5 samples<br>1/4: 6 samples<br>1/8: 7 samples<br>1/16: 6 samples	One-way ANOVA<br><br>Dunnett's multiple comparisons test	F(5,28) = 13.19, ****p < 0.0001<br><br>Control vs. 1/1: ****p < 0.0001<br>Control vs. 1/2: ***p = 0.0001<br>Control vs. 1/4: **p = 0.0017<br>Control vs. 1/8: p = 0.0273<br>Control vs. 1/16: p = 0.3276
Figure 3d	n = 4 brain sections	Paired t-test (two-tailed)	t(3) = 5.535, *p = 0.0116

Figure 3e	n = 16 brain sections<br><br>1/1: 4 samples<br>3/5: 4 samples<br>2/5: 4 samples	One-way ANOVA<br><br>Tukey's multiple comparisons test	F(2,9) = 6.164, *p = 0.021<br><br>1/1 vs. 3/5: p = 0.1747<br>1/1 vs 2/5: *p = 0.0166<br>3/5 vs. 2/5: p = 0.3233
Figure 4d	n = 26 brain samples<br><br>Control-EV: 13 samples<br>Shank3-cKO-EV: 13 samples	Unpaired t-test (two-tailed)	t(24) = 2.336, *p = 0.0206
Figure 4f	n = 69 brain sections<br><br>1/64: 30 sections<br>1/128: 25 sections<br>Control: 14 sections	One-way ANOVA<br><br>Dunnett's multiple comparisons test	F(2,66) = 7.073, **p = 0.0016<br><br>Control vs. 1/64: ***p = 0.0007<br>Control vs. 1/128: p = 0.0554
Figure 4g	n = 110 mice<br><br>1/1: 9 mice<br>1/2: 10 mice<br>1/4: 12 mice<br>1/8: 11 mice<br>1/16: 5 mice<br>1/32: 6 mice<br>1/64: 5 mice<br>1/128: 15 mice<br>1/256: 11 mice<br>1/512: 12 mice<br>Control: 14 mice	Two-way mixed model ANOVA	(Familiarity×Group)<br>F(10,99) = 1.937; *p = 0.0488<br>(Familiarity)<br>F(1,99) = 7.530; **p = 0.0072<br>(Group)<br>F(10,99) = 0.8769; p = 0.5573<br><br>1/1: F(1,8) = 0.034, p = 0.857<br>1/2: F(1,9) = 0.563, p = 0.472<br>1/4: F(1,11) = 0.052, p = 0.823<br>1/8: F(1,10) = 0.260, p = 0.621<br>1/16: F(1,4) = 0.033, p = 0.865<br>1/32: F(1,5) = 0.144, p = 0.720<br>1/64: F(1,4) = 0.057, p = 0.824<br>1/128: F(1,14) = 4.595, p = 0.050<br>1/256: F(1,10) = 4.698, p = 0.050<br>1/512: F(1,11) = 15.52, **p = 0.002<br>Control: F(1,13) = 7.759, *p = 0.015
Figure 4h	n = 110 mice<br><br>1/1: 9 mice<br>1/2: 10 mice<br>1/4: 12 mice<br>1/8: 11 mice<br>1/16: 5 mice<br>1/32: 6 mice<br>1/64: 5 mice<br>1/128: 15 mice	One-way ANOVA	F(10,99) = 2.009, *p = 0.0401

	1/256: 11 mice<br>1/512: 12 mice<br>Control: 14 mice		

Supplementary Table 1. Statistical information for Figures.

Supplementary Table 2		
**Name**	**Primer**	**Sequence (5’-3’)**
**Genotyping**		
Drd1-EGFP	Forward	5’-CCT ACG GCG TGC AGT GCT TCA GC-3’
	Reverse	5’-CGG CGA GCT GCA CGC TGC GTC CTC-3’
Shank3-KO	Forward	5’-GAG CTC TAC TCC CTT AGG ACT T-3’
	Reverse-1	5’-TCC CCC TTT CAC TGG ACA CCC-3’
	Reverse-2	5’-TCA GGG TTA TTG TCT CAT GAG C-3’
**AAV-mediated CRISPR/Cas9 delivery**		
Shank3-sgRNA-1	Forward	5’-CAC CGG CAG GGG CGT GTC CAG GTT AG-3’
	Reverse	5’-AAA CCT AAC CTG GAC ACG CCC CTG CC-3’
Shank3-sgRNA-2	Forward	5’-ACC AGG GGC GTG TCC AGG TTA GG-3’
	Reverse	5’-AAC CCT AAC CTG GAC ACG CCC CT-3’
EGFP-sgRNA-3	Forward	5’-ACC GGG CGA GGA GCT GTT CAC CG-3’
	Reverse	5’-AAC CGG TGA ACA GCT CCT CGC CC-3’
**EV-mediated CRISPR/Cas9 delivery**		
EGFP-EV#1	Forward	5’-CAC CGA CCA GGA TGG GCA CCA CCC-3’
	Reverse	5’-AAA CGG GTG GTG CCC ATC CTG GTC-3’
EGFP-EV#2	Forward	5’-CAC CGA GCT GGA CGG CGA CGT AAA-3’
	Reverse	5’-AAA CTT TAC GTC GCC GTC CAG CTC-3’
EGFP-EV#3	Forward	5’-CAC CGG GCG AGG AGC TGT TCA CCG-3’
	Reverse	5’-AAA CCG GTG AAC AGC TCC TCG CCC-3’
EGFP-EV#4	Forward	5’-CAC CGG CCA CAA GTT CAG CGT GTC-3’
	Reverse	5’-AAA CGA CAC GCT GAA CTT GTG GCC-3’
Shank3- cKO-EV	Forward	5’-CAC CGG GTG TAA ACT CCT CAA TGG-3’
	Reverse	5’-AAA CCC CAC ATT TGA GGA GTT ACC-3’
**Quantitative RT-PCR (qRT-PCR)**		
Actb	Forward	5’-GAC CCA GAT CAT GTT TGA GAC C-3’
	Reverse	5’-GCT GTG GTG GTG AAG CTG TA-3’
Shank3	Forward	5’-AAG CAG AGA CCC CCA TTA G-3’
	Reverse	5’-CAG TTC GAA GTC CAG CCC TC-3’

Supplementary Table 2. List of primer sequences used in the study.
