# Supplementary material for: Conditional knockout of Shank3 in the ventral CA1 by quantitative in vivo genome-editing impairs social memory in mice
Source: Nat Commun. 2024 Jun 12;15:4531. doi: 10.1038/s41467-024-48430-x (PMC11169449; doi:10.1038/s41467-024-48430-x)
Supplement: Supplementary file 3 — Reporting Summary [file 41467_2024_48430_MOESM3_ESM.pdf]

Corresponding author(s): Teruhiro Okuyama

Last updated by author(s): Apr 9, 2024

Reporting Summary

Nature Portfolio wishes to improve the reproducibility of the work that we publish. This form provides structure for consistency and transparency in reporting. For further information on Nature Portfolio policies, see our Editorial Policies and the Editorial Policy Checklist.

Statistics

For all statistical analyses, confirm that the following items are present in the figure legend, table legend, main text, or Methods section.

n/a	Confirmed	
☐	☒	The exact sample size (*n*) for each experimental group/condition, given as a discrete number and unit of measurement
☐	☒	A statement on whether measurements were taken from distinct samples or whether the same sample was measured repeatedly
☐	☒	The statistical test(s) used AND whether they are one- or two-sided<br>*Only common tests should be described solely by name; describe more complex techniques in the Methods section.*
☐	☒	A description of all covariates tested
☐	☒	A description of any assumptions or corrections, such as tests of normality and adjustment for multiple comparisons
☐	☒	A full description of the statistical parameters including central tendency (e.g. means) or other basic estimates (e.g. regression coefficient) AND variation (e.g. standard deviation) or associated estimates of uncertainty (e.g. confidence intervals)
☐	☒	For null hypothesis testing, the test statistic (e.g. *F*, *t*, *r*) with confidence intervals, effect sizes, degrees of freedom and *P* value noted<br>*Give P values as exact values whenever suitable.*
☒	☐	For Bayesian analysis, information on the choice of priors and Markov chain Monte Carlo settings
☒	☐	For hierarchical and complex designs, identification of the appropriate level for tests and full reporting of outcomes
☒	☐	Estimates of effect sizes (e.g. Cohen's *d*, Pearson's *r*), indicating how they were calculated

Our web collection on statistics for biologists contains articles on many of the points above.

Software and code

Policy information about availability of computer code

Data collection	EthoVision XT 15(Nordus)<br>FV3000 (Olympus)<br>BZ-X710 (Keyence)<br>FACS Aria III cell sorter (BD Biosciences)<br>Light Cycler 480 (Roche)
Data analysis	EthoVision XT 15 (Nordus)<br>ImageJ2 2.9.0 (NIH)<br>Prism 9.1.2 (GraphPad)<br>BD FACSDiva Software (BD Biosciences)<br>Prism 9 and 10 (GraphPad)<br>MATLAB R2021a (MathWorks)

For manuscripts utilizing custom algorithms or software that are central to the research but not yet described in published literature, software must be made available to editors and reviewers. We strongly encourage code deposition in a community repository (e.g. GitHub). See the Nature Portfolio guidelines for submitting code & software for further information.

Data

Policy information about availability of data

All manuscripts must include a data availability statement. This statement should provide the following information, where applicable:

- Accession codes, unique identifiers, or web links for publicly available datasets
- A description of any restrictions on data availability
- For clinical datasets or third party data, please ensure that the statement adheres to our policy

The source data underlying all Figures are provided as Source Data files. All data reported in this study will be shared by the lead contact upon request.

Human research participants

Policy information about studies involving human research participants and Sex and Gender in Research.

Reporting on sex and gender	Not applicable to this study
Population characteristics	Not applicable to this study
Recruitment	Not applicable to this study
Ethics oversight	Not applicable to this study

Note that full information on the approval of the study protocol must also be provided in the manuscript.

Field-specific reporting

Please select the one below that is the best fit for your research. If you are not sure, read the appropriate sections before making your selection.

☒ Life sciences ☐ Behavioural & social sciences ☐ Ecological, evolutionary & environmental sciences

For a reference copy of the document with all sections, see nature.com/documents/nr-reporting-summary-flat.pdf

Life sciences study design

All studies must disclose on these points even when the disclosure is negative.

Sample size	Suitable sample sizes were determined based on the Cohen's d effect size and our previous study as well as other's similar studies which are generally employed in the field of study: Okuyama et al., Science (2016), Hitti and Siegelbaum (2014).
Data exclusions	All mice used for AAV-mediated conditional knockout experiments were perfused and blindly post-hoc verified to include only individuals with appropriate expression for further analysis.
Replication	All behavioral experiments were conducted in at least three different batches and all batches showed a similar trend. All the cell culture experiments were conducted in at least two different batches and all batches showed a similar trend. All fluorescent image analyses were independently repeated at least twice and consistently demonstrated a similar trend.
Randomization	All the subject mice were randomly assigned to each experimental groups in each study. All the cell culture experiments were randomly assigned in each study.
Blinding	All the behavioral experiments were conducted with a blind group allocation during data collection and analysis. All animal behaviors were automatically tracked using EthoVision XT (Nordus). The cell culture experiments were not essentially not blinded because it was necessary for experimenters to be aware of the information of conditions to be compared.

Reporting for specific materials, systems and methods

We require information from authors about some types of materials, experimental systems and methods used in many studies. Here, indicate whether each material, system or method listed is relevant to your study. If you are not sure if a list item applies to your research, read the appropriate section before selecting a response.

Materials & experimental systems

n/a	Involved in the study
☐	☒ Antibodies
☐	☒ Eukaryotic cell lines
☒	☐ Palaeontology and archaeology
☐	☒ Animals and other organisms
☒	☐ Clinical data
☒	☐ Dual use research of concern

Methods

n/a	Involved in the study
☒	☐ ChIP-seq
☐	☒ Flow cytometry
☒	☐ MRI-based neuroimaging

Antibodies

Antibodies used

Primary antibodies: chicken anti-GFP antibody (1:1000, A10262, Thermo Fisher Scientific), rabbit anti-RFP antibody (1:1000, 600-401-379, Rockland), rabbit anti-Shank3 antibody (64555, 1:500; Cell Signaling Technology)
Secondary antibodies: anti-chicken Alexa Fluor-488 conjugated secondary antibody (1:500, A11039, Thermo Fisher Scientific), anti-rabbit Alexa Fluor-546 conjugated secondary antibody (1:500, A11010, Thermo Fisher Scientific), anti-rabbit Alexa Fluor-488 conjugated secondary antibody (1:500, A11008, Thermo Fisher Scientific)

Validation

The antibodies used in this study are validated by the manufacturers as shown in the following websites.
chicken anti-GFP antibody: https://www.thermofisher.com/antibody/product/GFP-Antibody-Polyclonal/A10262
rabbit anti-RFP antibody: https://www.rockland.com/categories/primary-antibodies/rfp-antibody-pre-adsorbed-600-401-379/
rabbit anti-Shank3 antibody: https://www.cellsignal.com/products/primary-antibodies/shank3-d5k6r-rabbit-mab/64555
anti-chicken Alexa Fluor-488 conjugated secondary antibody: https://www.thermofisher.com/antibody/product/Goat-anti-Chicken-IgY-H-L-Secondary-Antibody-Polyclonal/A-11039
anti-rabbit Alexa Fluor-546 conjugated secondary antibody: https://www.thermofisher.com/antibody/product/Goat-anti-Rabbit-IgG-H-L-Cross-Adsorbed-Secondary-Antibody-Polyclonal/A-11010
anti-rabbit Alexa Fluor-488 conjugated secondary antibody: https://www.thermofisher.com/antibody/product/Goat-anti-Rabbit-IgG-H-L-Cross-Adsorbed-Secondary-Antibody-Polyclonal/A-11008

Eukaryotic cell lines

Policy information about cell lines and Sex and Gender in Research

Cell line source(s)

HEK293T: Takara (cat. no. 632617)
HEK293-EGFP: GenTarget Inc (SC001)
NIH-3T3: Riken BioResource Research Center

Authentication

HEK293-EGFP cell line was purchased directly from the provider. HEK293T and NIH-3T3 cell lines were gifted from Dr. A. Miyajima (IQB, The University of Tokyo). No additional authentication was performed by the authors.

Mycoplasma contamination

The cell lines were not tested for mycoplasma contamination.

Commonly misidentified lines
(See ICLAC register)

Commonly misidentified cell line was not used

Animals and other research organisms

Policy information about studies involving animals; ARRIVE guidelines recommended for reporting animal research, and Sex and Gender in Research

Laboratory animals

C57BL/6J (B6), C3H/HeJ, and BALB/c male mice were obtained from Clea Japan. Drd1-EGFP (Tg(Drd1-EGFP)X60Gsat/Mmmh, RRID: MMRRC_000297-MU) was obtained from the Mutant Mouse Resource and Research Center. Shank3-KO (B6.129-Shank3tm2Gfng/J, RRID: IMSR_JAX:017688) was obtained from The Jackson Laboratory. All animals were housed in the Institute for Quantitative Biosciences facility under a 12 h (7 am – 7 pm) light/dark cycle, 23 ± 2 °C, 50 % humidity with food and water ad libitum. Adult male B6 mice (3–5 months old) were used as subjects for behavioral assays. Juvenile male C3H/HeJ and BALB/c mice (5–8 weeks old) were used as demonstrators. Adult male Drd1-EGFP mice (3–5 months old) were used for the in vivo confirmation of EV-mediated Cas9/sgRNA RNP delivery. Adult male Shank3-KO (3–5 months old) mice were used for Shank3 antibody confirmation. Juvenile male B6 mice (7 weeks old) were used for social memory engram neuron labeling experiment.

Wild animals

No wild animals were used in this study.

Reporting on sex

Only male mice were used in this study.

Field-collected samples

No field-collected samples were used in this study.

Ethics oversight

All procedures were performed in accordance with protocols approved by the Institutional Animal Care and Use Committee at the Institute for Quantitative Biosciences, the University of Tokyo (Protocol number 2915 (2018), 3112 (2019), 0201 (2020), 0314 (2021), 0403-2 (2022), A2022IQB018 (2023)).

Note that full information on the approval of the study protocol must also be provided in the manuscript.

Flow Cytometry

Plots

Confirm that:

☒ The axis labels state the marker and fluorochrome used (e.g. CD4-FITC).

☒ The axis scales are clearly visible. Include numbers along axes only for bottom left plot of group (a 'group' is an analysis of identical markers).

☒ All plots are contour plots with outliers or pseudocolor plots.

☒ A numerical value for number of cells or percentage (with statistics) is provided.

Methodology

Sample preparation	HEK293-EGFP cells were washed twice with 300 µl of PBS (-) and incubated with 500 µl of trypsin at 37°C for 3 min. Then, 500 µl of 3% FBS/PBS (-) was added to each well and plates were centrifuged at 600 g for 8 min at RT in a sterile 1.5 ml tube to pellet the cells. The cell pellets were suspended in 1 ml of 3% FBS/PBS (-), and the suspended solution was filtered through a 35-µm cell strainer (Falcon) to remove aggregated cells.
Instrument	FACS Aria III cell sorter (BD Biosciences)
Software	BD FACSDiva Software
Cell population abundance	10,000 or 50,000 HEK293-EGFP cell events were collected for each samples
Gating strategy	FSC/SSC gates were employed to eliminate potential doublet cells, ensuring the inclusion of only singlet cells.

☒ Tick this box to confirm that a figure exemplifying the gating strategy is provided in the Supplementary Information.
